# Level of immunization coverage and associated factors among children aged 12–23 months in Lay Armachiho District, North Gondar Zone, Northwest Ethiopia: a community based cross sectional study

**DOI:** 10.1186/s13104-015-1192-y

**Published:** 2015-06-13

**Authors:** Melkamu Beyene Kassahun, Gashaw Andargie Biks, Alemayehu Shimeka Teferra

**Affiliations:** Armachiho District Health Office, North Gondar Zone, Amhara Regional State Ethiopia; Department of Health Service Management and Health Economics, College of Medicine and Health Sciences, University of Gondar, Gondar, Ethiopia; Department of Epidemiology and Biostatistics, College of Medicine and Health Sciences, University of Gondar, P.O. Box 196, Gondar, Ethiopia

**Keywords:** Immunization coverage, Lay Armachiho district, Children aged 12–23 months, Ethiopia

## Abstract

**Background:**

Immunization against childhood disease is one of the most important public health interventions with cost effective means to preventing childhood morbidity, mortality and disability. However, complete immunization coverage remains low particularly in rural areas of Ethiopia. This study aimed to assess the level of immunization coverage and associated factors in Lay Armachiho District, North Gondar zone, Northwest Ethiopia. A community based cross-sectional study was conducted in March, 2014 among 751 pairs of mothers to children aged 12–23 months in Lay Armachiho District. A two stage sampling technique was employed. Logistic regression analysis was carried out to compute association between factors and immunization status of children. Backwards stepwise regression method was used and those variables significant at *p* value 0.05 were considered statistically significant.

**Results:**

Seventy-six percent of the children were fully immunized during the study period. Dropout rate was 6.5% for BCG to measles, 2.7% for Penta1 to Penta3 and 4.5% for Pnemonia1 to Pnemonia3. The likelihood of children to be fully immunized among mothers who identified the number of sessions needed for vaccination were higher than those who did not [AOR = 2.8 (95% C1 = 1.89, 4.2)]. Full immunization status of children was higher among mothers who know the age at which the child become fully immunized than who did not know [AOR = 2.93 (95% CI = 2.02, 4.3)]. Taking tetanus toxoid immunization during pregnancy showed statistically significant association with full immunization of children [AOR 1.6 (95% CI = 1.06, 2.62)]. Urban children were more likely to be fully immunized than rural [AOR = 1.82 (95% CI = 1.15, 2.80)] and being male were more likely to be fully immunized than female [AOR = 1.80 (95% CI = 1.26, 2.6)].

**Conclusion and recommendation:**

Vaccination coverage was low compared to the Millennium Development Goals target. It is important to increase and maintain the immunization level to the intended target. Efforts should be made to promote women‘s’ awareness on tetanus toxoid immunization, when the child should start vaccination, number of sessions needed to complete immunization, and when a child become complete vaccination to improve immunization coverage through health development army and health professionals working at antenatal care, postnatal care and immunization units.

## Background

Vaccination has been shown to be one of the most cost effective health interventions worldwide, through which a number of serious childhood diseases have been successfully prevented or eradicated. The immunization campaign carried out from 1967 to 1977 by the World Health Organization (WHO) eradicated the natural occurrence of small pox and currently polio has been eradicated from the Americas (in 1994), the Western Pacific (in 2000), and the European World Health Organization (WHO) regions (in 2002) [1].

Since the commencement of the Expanded Program on Immunization in 1974, the widespread use of vaccines has substantially reduced vaccine preventable morbidity and mortality worldwide; however, a significant proportion of children are not being fully vaccinated for the recommended vaccines [2, 3].

The World Health Organization (WHO) recommends vaccines including diphtheria, tetanus, pertussis, hepatitis-B, invasive hemophilus influenza B, and measles vaccine for all children. However, many infants and children still die every year from these diseases. In 2011, nearly 107 million infants (83%) worldwide received at least 3 doses of DTP vaccine. Although, approximately 22.4 million failed to receive 3 doses leaving large numbers of children susceptible to vaccine preventable diseases and death. As a result 2–3 million children are become ill and dying annually [4].

In the 2011 WHO estimate of the global DPT3 coverage among children aged <12 months, it was 85% ranging from 71% in African region to 96% in western pacific [5]. Despite substantial progress continues to be made, many children particularly those in less developed countries remain at risk of vaccine preventable diseases which makes achieving high and equitable coverage a challenge in low income countries like Ethiopia [3, 6].

In Ethiopia, vaccination is being given on routine and outreach basis. The routine vaccination schedule recommends that infants should be vaccinated starting from birth and complete their vaccination before 1 year of life with one dose bacillus Calmette–Guerin (BCG) at birth or as soon as possible and oral polio vaccine initial doze (OPV_0_). Three doses of OPV, Pentavalent, Rota1, Rota2 and pneumonia vaccines are given at interval of 4 weeks duration at the 6th, 10th and 14th weeks, respectively and finally measles vaccine is given at the age of 9 months. A child is said to be fully vaccinated if he/she received all vaccines according to the schedule [6].

Ninety percent of mortality in under-five children in Ethiopia is caused by pneumonia, malaria, diarrhea, measles and neonatal causes such as pre-maturity, asphyxia and neonatal sepsis. Malnutrition and HIV are the two underlying conditions attributing to 57 and 11% of these deaths, respectively.

In Ethiopia, about 1 million children were estimated to be unvaccinated [7] and 16% under-five mortality has been attributed to vaccine preventable diseases [8]. Immunization is one of the national child survival strategies in the country targeted to achieve diphtheria-pertussis and tetanus (DPT3)/measles vaccination coverage of 90% in 2010 [2].

Child immunization is the key to achieving the millennium development goals (MDGs) especially to reduce child morbidity and mortality. The proportion of children immunized against measles is one of the indicators of MDG4 [9]. However in Ethiopia, the incidence of measles has increased from 3.19 per 100,000 in 2009 to 7.35 per 100,000 in 2010 with a total of confirmed 1,964 and 3,121 cases, respectively [10].

According to the Ethiopian Demographic and Health Surveys (EDHS) 2011 report, overall 24% of children aged 12–23 months were fully vaccinated at the time of the survey. Although this indicated a 19% increase from the level reported in the 2005 EDHS report, the percentage of children who were fully vaccinated remains far below the goal of 66% set in the national Health Sector Development Program four (HSDP IV). The Ethiopia Demographic and Health Survey is part of the worldwide MEASURE DHS project which is conducted every 5 years and funded by the United States Agency for International Development (USAID). There is a wide variation among regions regarding full immunization coverage ranging from 79% in Addis Ababa to 9% in Afar region [6, 11, 12]. In order to improve vaccination coverage, multifaceted and tailored strategies will be required to address factors contributing to incomplete immunization, particularly in countries with the large numbers of unvaccinated children. As research evidences indicated, low access to services, inadequate awareness on immunization, missing opportunities, inconvenient immunization schedules, distance from health facilities, fear of vaccine side effects are the major factors contributing to high dropout rate and low immunization coverage in the country [2, 5, 11]. In preventing the development of vaccine preventable diseases, achieving and maintaining high level immunization coverage must therefore be a priority to the health systems through different approaches. Amhara was one of the regions with low immunization coverage (26.7%) compared to other national regional states such as Addis Ababa and Tigray 82.5% and 60.8%, respectively. There was no study that had assessed the level of immunization coverage and factors associated with it in the study area. Therefore, the aim of this study was to determine the level of complete immunization coverage and factors associated with it among all pairs of mothers to children aged 12–23 months in Lay Armacheho District, North Gondar Zone, Amhara regional state, Northwest Ethiopia.

## Methods

### Study design and setting

A community based cross sectional study was conducted in Lay Armacheho district, Northwest Ethiopia from March 01 to 31, 2014. The district is found is Amhara National regional state, North Gondar zone. The district has one urban and thirty three rural kebeles^a^. Tekele Dengay is the capital of the district, which is 26 km away from Gondar city. According 2007 census, the district has a total projected population of 178, 209 of which 89, 995 and 88, 214 were females and males, respectively [11]. The town has telephone service as well as 24 h electricity. The district has eight government health centers, 34 health posts^b^, and eight private clinics. Geographical health services coverage of the district was 100% [12].

### Sample size and sampling procedure

The required sample size was determined using single population proportion formula. The following assumptions were made during calculating the sample; full immunization coverage of children under 1 year of age from EDHS 2011 which was 24, 95% confidence level, margin of error 5 and 10% non response rate and design effect of 2. The sample size was calculated to be 617. However, when factor analysis was done considering factors from previous studies such knowledge of mothers on vaccination (79%), delivering at health facility (10%) and following antenatal care (34%), we found a sample size of 550, 304 and 757, respectively. Among the all the calculated samples, the largest was 757. Finally, the largest sample size was taken for this study [13].

### Sampling procedure

Two stage sampling technique was employed. At the first stage, from 34 kebeles 20% of the kebeles were considered to be included in the study. From the total rural kebeles six were selected randomly by lottery method. There was one urban kebele in the district and it was taken purposively to represent urban population. Then at the kebeles level, individual households were selected using systematic random sampling technique. The sample interval of the households in each kebele was determined by dividing the total number of households to the allocated sample size. The sample size was distributed to each kebele proportional to the size of households the kebeles. Children in the selected households were further selected. If there were two or more children in the same household, lottery methods was used to select only one. When there was no eligible child in the selected household, the next households were included in the study. If eligible participants were not found at home during data collection, interviewers have revisited the households for two consecutive times and when the interviewers failed to find the eligible participant after two visits, the next household was included for the study.

### Operational definition

#### Fully vaccinated

A child 12–23 months old who received one dose of BCG, three dose of pentavalent, three dose of OPV, three dose of hemophilus influenza B and one dose of measles before the child’s first birthday and measured by vaccination card and mothers recall.

### Data collection instrument and procedures

Structured and pre-tested questionnaire first prepared in English and translated to Amharic was used to collect data. Data was collected by fourteen diploma nurses recruited from the district. Face to face interview was conducted to collect data about immunization from mothers or care givers of the eligible child in the selected households. First, the mother or care giver was asked whether the child does have vaccination card. If she replied “yes”, she was requested to bring the vaccination card and data collectors have to observe all the types of vaccines (BCG, OPV, pentavalent, measles vaccine with Vit-A) the child received according as to the schedule checking the date, month, and year. If the card indicated this information, the child was labeled as fully immunized otherwise not fully immunized. If the mother or care giver replied “there is no card”; it may be lost, unable to access at the time of data collection or some other reason, other probing questions and techniques were considered to confirm that the child was fully immunized. For instance, for BCG vaccine, the mothers or care takers were asked that their children were given BCG vaccine against tuberculosis that is an injection in the right shoulder and observation of BCG scar at the right arm.

For polio vaccine, the mother or care giver was asked as “was the child given polio vaccine that is dropped in mouth?” If she replied “yes”, “how many times polio vaccine was given?” Similarly for pentavalent vaccines, the mother or care giver was asked as “was the child given pentavalent vaccine, an injection given on right thigh or buttocks?” If she replied “yes”, how many times the child was given? In addition for measles, the mother or care giver was asked as “was the child given measles vaccine in the thigh or buttocks at age of 9 months?” Such questions were considered to confirm that the child has taken complete immunization as to the recommended age.

### Data quality control

Data quality was controlled through provision of training to the data collectors and supervisors about the overall data collection procedures and the techniques of interviewing. Pre-test was done using 5% of participants in one kebele which was not actually included in the main study. After conducting pre-test, the necessary corrective modifications were made. The collected data was checked for completeness, consistency, accuracy and clarity by the supervisors and the principal investigator on a daily base.

### Data processing and analysis

The collected data was coded, entered and cleaned using Epi info version 7 and then exported into SPSS version 20 for analysis. Child immunization status was dichotomized into two: fully immunized if a child is completed all the required vaccines as to the schedule and not fully immunized if a child missed any dose of the vaccine. Bi-variable and multivariable analyses were carried out to assess the association between factors and immunization status of children. All variables with p value of 0.2 and below in the bi-variable analysis were entered into multiple logistic regression model. Finally, back ward stepwise regression method was used and those variables having p value 0.05 and below were considered to have statistically significant association with the complete child immunization.

## Ethical approval

Ethical clearance was obtained from the Institutional Review Board of Institution of Public Health, University of Gondar. Permission letter was obtained from Lay Armacheho District administrative office. After brief explanation of the objectives and purpose of the study, verbal informed consent was obtained from each study participant. Participants were also informed that participation was on voluntary basis and they have the right to stop their participation at any time. Study participants were also informed that all data obtained from them would be kept confidential by using codes instead of any personal identifiers.

## Results

### Socio-demographic characteristics of children and mothers

A total of 751 pairs of mothers and child aged 12–23 months old were interviewed with response rate of 99.2%. The age of the mothers in this study was ranged from 18 to 46 years with mean and median of 27.2 and 26 years, respectively. Half of the mothers were not able to read and write (50%). More than ninety percent of the respondents were married (91.3%). About ninety percent (89.3%) of the mothers were Orthodox Christian and 10.7% were Muslims. Nearly 60% of the households have family size of 4 and below and the rest 41% have more than four family members. Seventy two percent of the respondents walk 30 min and less to reach the nearest health facility. Three hundred eighty four (51%) of children were males and 49% were females (Table 1).

**Table 1:** Socio-demographic characteristics of children and mothers in Lay Armachiho District, North Gondar Zone, Northwest Ethiopia, 2014 (n = 751)

Variables	Frequency	%
Sex of children		
Male	384	51.0
Female	367	49.0
Mothers age in years		
<20	118	15.5
20–34	474	63.3
35–49	159	21.2
Educational status		
Not read and write	376	50.1
Read and write	14	1.9
Grade 1–8	205	27.3
Grade 9 and above	156	20.7
Marital status		
Married	686	91.3
Divorced	50	6.7
Not married	7	0.9
Separated	5	0.7
Widowed	3	0.4
Mother’s occupation		
Farmer	21	2.8
Housewife	608	81.0
Govt. employee	37	4.9
Merchant	39	5.2
Daily laborer	22	2.9
Private sectors	14	1.9
Student	10	1.3
Ethnicity		
Amhara	404	53.8
Kemant	347	46. 2
Religion		
Orthodox	671	89.5
Muslim	80	10.5
Monthly income		
≤2,249	354	47.1
2,250–3,818	177	23.6
≥3,819	220	29.3
Family size		
≤4	442	58.9
≥5	309	41.1
Time to reach the nearest health facility		
<15 min	321	42.7
15–30 min	218	29.0
30–60 min	181	24.1
>60 min	31	4.1

### Maternal and children characteristics

Seventy seven percent of the mothers have attained antenatal care at least once during their pregnancy of the study children. Thirty five percent of them have three and more ANC visits. In addition, 79.5% of mothers took one or more dose of tetanus toxoid vaccine (Table 2).

**Table 2:** Maternal and child characteristics in Lay Armachiho District, North Gondar Zone, Northwest Ethiopia, 2014

Variables	Frequency	%
ANC follow up		
Yes	582	77.0
No	169	23.0
Number of antenatal received (582)		
≤2	379	65.0
≥3	203	35.0
Tetanus toxoid immunization received		
Yes	597	79.5
No	154	20.5
Number of TT immunization received (630)		
≤2	239	38.0
≥3	391	62.0
Postnatal care received		
Yes	218	29.0
No	533	71.0
Number of postnatal care received (218)		
≤2	122	56.0
≥3	96	44.0
Place of birth for the child		
Health facility	241	32.0
Home	510	68.0
Birth order of the child		
First	267	35.6
Second	179	23.8
Third	94	12.5
Fourth and above	211	28.1

### Mothers awareness on vaccine

Almost all of the study participants were heard of vaccination. Ninety one percent of them heard from health professionals and 25.8% from mass media. Of the total respondents, 27% of them stated that vaccination for a child starts at birth and 73% of them don’t know the age at which the child begins vaccination (Table 3).

**Table 3:** The respondent’s awareness on vaccination in Lay Armacheho District, North Gondar Zone, Northwest Ethiopia, 2014

Variables	Frequency	%
Heard about vaccination		
Yes	745	99.2
No	6	0.8
Source of information		
Health workers	686	66.8
Radio	194	18.9
Television	51	4.9
Friends	39	3.8
School	57	5.6
Able to know when to start vaccination		
Yes	203	27.0
No	548	73.0
Vaccination for a child is started		
Just after birth	203	27.0
One month later	492	65.4
Any time	10	1.3
I don’t know	46	6.2
Vaccination sessions needed		
Once	4	0.5
Twice	19	2.5
Three times	312	41.5
Five times	308	40.9
I don’t know	108	14.4
Able to know at what age to complete vaccination		
Yes	519	69.0
No	232	31.0
When to complete vaccination?		
9 months	519	69.1
1 year	103	13.7
5 years	65	8.7
I don’t know	30	4.0
Others^a^	34	4.5

^a^Includes up to 18 age and any time in life.

### Immunization coverage by card plus recall method

Among the total, 76.03% of children were fully immunized based on vaccination card and mothers recall method. More than eighty five percent (85.5%) of children took OPV1 vaccine followed by BCG 81.4% as to the schedule. Seventeen (2.3%) of children had not received any vaccination. Dropout rate, the proportion of children who started a certain vaccine but not complete the next intended vaccine was 5.2% for BCG to measles, 2.7% for pentavalent 1 to pentavalent 3 and 4.5% for PCV1 to PCV3 (Table 4).

**Table 4:** Vaccination Coverage of children aged 12–23 months based on child vaccination card and mothers recall, in Lay Armachiho District, North Gondar Zone, Northwest Ethiopia, 2014

Vaccines	Coverage by card		Coverage by recall		Coverage both by card and recall	
	Frequency	%	Frequency	%	Frequency	%
BCG	228	30.4	383	50.9	611	81.4
OPV1	244	32.5	398	52.9	642	85.5
OPV2	234	31.2	376	50.0	610	81.0
OPV3	218	29.0	374	49.8	592	78.8
Pentavalent1	237	31.6	371	49.4	608	80.9
Pentavalent2	230	30.6	368	49.0	598	79.6
Pentavalent3	216	28.8	365	48.6	581	77.4
Pnemonia1	212	28.2	391	52.0	603	80.3
Pnemonia2	202	26.9	390	51.9	592	78.8
Pnemonia3	191	25.4	388	51.7	576	76.7
Measles	189	25.2	390	51.9	579	77.0
Fully vaccinated	185	24.9	386	51.4	571	76.0

### Reasons for not fully vaccinated

**Figure 1:**
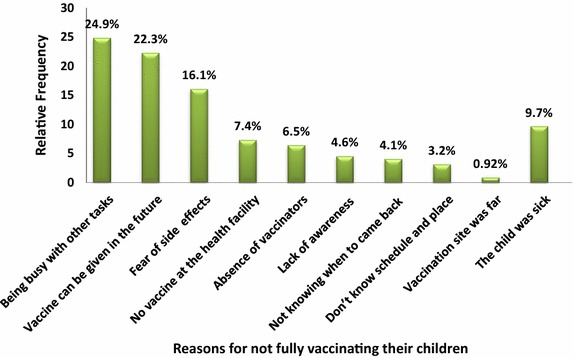
Reasons for not fully vaccinating children in Lay Armachiho District, North Gondar Zone, Northwest Ethiopia, 2014.

For those children who were not fully immunized, mothers were asked reasons for not completing their vaccination. Out of the total children who were not fully immunized, 24.9% of the mothers said that being busy in other tasks and 22.3% of them stated vaccines can be given in the future regardless of the normal schedule (Figure 1).

### Factors associated with full immunization status of children

Logistic regression analysis was used to assess the presence of association between the outcome and independent variables. On the bi-variable analysis, educational status, ethnicity, antenatal care follow up, number of antenatal care visits, having tetanus toxoid immunization, frequency of tetanus toxoid vaccine, postnatal care, sex of the child, place of delivery, mothers’ knowledge on the advantage of vaccination, mothers knowledge of sessions needed to complete vaccination and mothers’ knowledge of the age when the child to be fully immunized were found to be significantly associated with full immunization status with P value of 0.05 and below.

In multivariable analysis, residence, mothers’ knowledge of sessions needed to complete vaccination, sex of the child, mothers’ knowledge of the age when the child to be fully immunized and having tetanus toxoid immunization were found to be significantly associated with the outcome variable with P value of 0.05 and below.

Children born from urban mothers were 1.8 times more likely to complete their immunization compared to those born from rural mothers [AOR 1.8 (95% CI = 1.15, 2.89)]. Mothers who were able to know the number of sessions needed for immunization were three times more likely to fully vaccinate their children than mothers who were not able to know the sessions of immunization [AOR = 2.8 (95% CI = 1.89, 4.17)]. Full immunization status of children was three times higher among mothers who were able to know the age at which a child will be fully immunized compared to those who were not able to know [AOR = 2.9 (95% CI = 2.02, 4.26)]. Male children were 1.8 times more likely fully immunized than females [AOR = 1.8 (95% CI = 1.26, 2.60)]. Mothers who took tetanus toxoid immunization during their pregnancy were 1.66 times more likely to fully vaccinate their children compared to mothers who did not take tetanus toxoid immunization [AOR 1.6 (95% CI = 1.06, 2.62)] (Table 5).

**Table 5:** Bi-variable and multi-variable analysis of factors associated with full immunization status of children in Lay Armachiho District, North Gondar Zone, 2014

Variables	Fully vaccinated		Odd ratio (95% CI)	
	Yes (%)	No (%)	Crude OR	Adjusted OR
Education status				
Can’t read and write	245 (73.1%)	90 (26.9%)	1	
Read and write	33 (57.9%)	24 (42.1%)	0.5 (0.28, 0.90)*	
Grade 1–8	164 (80.4%)	40 (19.6%)	1.5 (0.98, 2.29)	
Grade 9 and above	129 (83.2)	26 (16.8%)	1.8 (1.12, 2.96)*	
Religion				
Orthodox	516 (76.9%)	155 (23.1%)	1	
Muslim	55 (68.8%)	25 (31.2%)	0.6 (0.39, 1.096)	
Ethnicity				
Amhara	320 (79.2%)	84 (20.8%)	1.4 (1.041, 2.039)*	
Kemant	251 (72.3%)	96 (27.7%)	1	
Residence				
Urban	138 (81.2%)	32 (18.8%)	1.4 (0.96, 2.26)	1.8 (1.15, 2.89)*
Rural	433 (74.5%)	148 (25.5%)	1	1
Gender				
Male	310 (80.7%)	74 (19.3%)	1.7 (1.21, 2.38)*	1.8 (1.26, 2.60)*
Female	261 (71.7%)	106 (28.9%)	1	1
Able to know when to start vaccination				
Yes	161 (79.3%)	42 (20.7%)	1.3 (0.87, 1.90)	
No	410 (74.8%)	138 (25.2%)	1	
Able to know session needed				
Yes	259 (84.4%)	48 (15.6%)	2.3 (1.57, 3.30)*	2.8 (1.89, 4.17)*
No	312 (70.3%)	132 (29.7%)	1	1
Able to know at what age to complete vaccination				
Yes	422 (81.3%)	97 (18.7%)	2.4 (1.71, 3.43)*	2.9 (2.02, 4.26)*
No	149 (64.2%)	83 (35.8%)	1	1
Place of delivery				
Health institution	198 (82.2%)	43 (17.8%)	1.691 (1.153, 2.482)*	
Home	373 (73.1%)	137 (26.9%)	1	
TT immunization				
Yes	493 (78.3%)	137 (21.7%)	1.9 (1.30, 3.01)*	1.6 (1.06, 2.62)*
No	78 (64.5%)	43 (35.5%)	1	1
ANC received				
Yes	453 (77.8%)	129 (22.2%)	1.5 (1.03, 2.22)*	
No	118 (69.8%)	51 (30.2%)	1	
PNC received				
Yes	176 (80.7%)	42 (19.3%)	1.4 (0.99, 2.15)*	
No	395 (74.1%)	138 (25.9%)	1	
Round of TT immunization				
None	106 (68.8%)	48 (31.2%)	1	
1–2	179 (74.9%)	60 (25.1%)	1.4 (0.86, 2.11)	
≥3	286 (79.9%)	72 (20.1%)	1.7 (1.17, 2.75)*	
Round of ANC				
None	118 (69.8%)	51 (30.2%)	1	
1–2	119 (77.3%)	35 (22.7%)	1.4 (0.89, 2.42)	
≥3	334 (78%)	94 (22%)	1.5 (1.02, 2.29)*	
Round of PNC				
None	395 (74.1%)	138 (25.9%)	1	
1–2	98 (80.3%)	24 (19.7%)	1.4 (0.87, 2.32)	
≥3	78 (81.2%)	18 (18.8%)	1.5 (0.87, 2.61)	

1.00 = Reference.

* p <=0.05 (significance).

## Discussion

This study tried to assess level of immunization coverage and associated factors among children aged between 12 and 23 months living in selected seven kebeles of Lay Armachiho district, which is a region near to Sudanese border in which measles outbreak is common. Full immunization status of children was confirmed using vaccination card of children and mothers recall method. This study found that the level of immunization coverage in the study area using either immunization card or maternal or care giver recall method was 76%. Coverage for each vaccine was 81.4% for BCG, 85.5% for OPV1, 81.0% for OPV2, 78.8% for OPV3, 80.9% for pentavalent1, 79.6% for pentavalent2, 78.7% for pentavalent3, 80.3% for pneumococcal conjugated vaccine (PCV1), 78.8% for PCV2, 76.7% for PCV3, and 77% for measles vaccine. According to the EPI schedule of Ethiopia, OPV and pentavalent vaccines are being given with similar schedule, however, OPV coverage was slightly higher than pentavalent which could be due to the fact that there are national immunization supplement campaigns for OPV and measles. Dropout rate from BCG to measles was higher than dropout rate from pentavalent1 to pentavalent3 and from PCV1 to PCV3. Because of the relatively longer interval between the third dose of pentavalent and measles, a number of children may not return for measles vaccine and this make the coverage rate for this antigen to be lower than other vaccines. However, this finding is higher compared to other studies such as EDHS 2011 and a study done in Ambo Woreda, central Ethiopia in 2011 [6, 14]. The possible explanation could be: EDHS 2011 has used samples from all the regions of Ethiopia including developing regions Afar, Somali, Benishangule Gumuz and Gambella. The residents of these regions are pastoral that may have low awareness and health seeking behaviour including vaccination of their children.

This study showed that there is decline in coverage of immunization from BCG (81.4%) at birth to measles (77.0%) and the proportion of fully immunized children (76.0%) which indicated that there was significant proportion of defaulting children.

Mother or care givers mention different reasons for not completing immunization to children. These reasons were being busy in other tasks during the immunization schedule, considering that the vaccines can be given in the future, fear of side effects of vaccines, and unavailability of vaccines in the health facility during the schedule. Findings from others studies have reported similar reasons with this finding [14].

Regarding factors associated with full immunization status of children, residence, mothers’ knowledge of sessions needed to complete child vaccination, sex of the child, mothers’ knowledge of the age at which the child to be fully immunized and having tetanus toxoid immunization were found to have significant association with the outcome variable. Urban children were 1.8 times more likely [(95% CI = 1.15, 2.80)] to complete their immunization compared to rural counterparts. This result is in agreement with a study done in Kenya [15].

In this study, maternal age does not have significant effect on immunization status of children. This finding is not consistent with a study done in rural Bangladesh 2010 which showed that age of the mother is an important factor for a child to be fully immunized. Mothers in the middle age group were more likely to fully immunize their children than the youngest and oldest age group [16].

Mothers who took tetanus toxoid immunization during their pregnancy were 1.66 times more likely to fully vaccinate their children compared to mothers who did not take tetanus toxoid immunization. This study had consistent result with study done in Bangladesh [17]. The possible explanation could be mothers who visited health facilities for tetanus toxoid immunization might have exposure to information about the importance of completing immunization.

Mothers who were able to identify the number of sessions needed for immunization were three times more likely to fully vaccinate their children compared to mothers who were not able to identify the number of sessions. Similarly, full immunization status of children was three times higher among mothers who were able to know the age at which a child will be fully immunized compared to those who were not able to know. This finding was found to be similar with the study done in Ambo woreda, central Ethiopia [18]. This may indicate that mothers who were able to know the number of sessions and the age at which a child will be fully immunized might have good awareness on completing child immunization during antenatal care follow up, through media or other sources of information. Male children were more likely to be fully immunized than females. This showed that there could be sexual discrimination between male and female children since in the Ethiopian culture particularly rural population, priority is given to males. This was similar with study done in Ambo district and Bangladesh in which female children were less likely to be fully immunized compared to male males [19].

## Limitation of the study

This study may have recall bias. Since the outcome and factors were assessed simultaneously, it may not be possible to establish cause effect relationship between outcome variable and associated factors.

## Conclusion

Level of immunization coverage was found to be low among children aged 12–23 months in lay study district compared to the national MDG target (at least 90.0%) to be achieved by 2015.

Mothers who took tetanus toxoid immunization during pregnancy, sex of the child, residence, mothers who were able to know the number of sessions needed and the age at which the child will complete vaccinations were found to be factors associated with full immunization status of children.

## Recommendation

Generally, the findings of this study have important policy implications for health intervention programs and underline the view that encouraging awareness of the community on the importance of the recommended immunization may have a considerable importance on childrens’ health. Therefore, the following recommendations were forwarded based the findings of this study.

- Efforts should be made to maintain and increase the current immunization coverage to reach the intended target.
- Supervision and monitoring of the rural population in routine should be strengthened and it would be better to arrange supplemental immunization activities particularly for defaulting children considering context of the rural community.
- Sufficient information should be provided to rural women, which will encourage them to seek immunization at the appropriate age of the child.
- Health extension workers and health development army should rigorously aware the mothers and the community about the advantage of completing immunization for children, when a child start vaccine, the number of sessions needed, and when a child will be fully immunized.
- There should be tracing mechanism and reminders to defaulting parents until their children complete the required vaccination as to the schedule with particular emphasis to the rural community.

## Endnotes

^a^Kebele is the smallest administrative unit of the Federal Democratic Republic of Ethiopia.

^b^Health post—the smallest unit of health facility at the lowest level of administration (kebele) providing basic health services to the community.

## Abbreviations

AOR: adjusted odds ratio
BCG: bacillus Calmette Guerin
DPT: diphtheria, pertusis and tetanus
OPV: oral polio vaccine
MDG: Millennium Development Goals
EDHS: Ethiopian Demographic and Health Surveys
Vit A: vitamin A
PCV: pneumococcal conjugated vaccine

## References

[1] Angela G, Zulfiqar B, Lulu B, Aly GS, Dennis JGR, Anwar H (2010). Pediatric disease burden and vaccination recommendations: understanding local differences [systematic review]. Int J Infect Dis.

[2] Odusanya OO, Alufohai JE, Meurice FP, Ahonkhai VI (2008). Determinants of vaccination coverage in rural Nigeria. BMC Public Health.

[3] World Health Organization. WHO morbidity and mortality weekly report 2012 November 11 Contract No.: No. 44

[4] World Health Organization. WHO morbidity and mortality weekly report 2012 November 11 Contract No.: No. 43

[5] Antai D (2010). Migration and child immunization in Nigeria: individual- and community-level contexts. BMC Public Health.

[6] Okwaraji YB (2012). The association between travel time to health facilities and childhood vaccine coverage in rural Ethiopia. A community based cross sectional study. BMC Public Health.

[7] Etana B, Deressa W (2012). Factors associated with complete immunization coverage in children aged 12–23 months in Ambo woreda, Central Ethiopia. BMC Public Health.

[8] Central Statistical Agency of Ethiopia (CSA). Ethiopia Demographic and Health Survey 2011

[9] World Health Organization (2009) WHO Global elimination of measles. Organization WH, Geneva

[10] Lulsegad S, Mekasha A, Berhane Y (2006) Common childhood disease. In: Berhane Y, Haile Mariam D, Helmut K (eds) Epidemiology and ecology of health and disease in Ethiopia, vol 329. Shama Books, Addis Ababa

[11] Federal Ministry of Finance and Economic Development (MOFED) (2012) Assessing millennium development goals, Ethiopia MDG report

[12] Birhanu AB, Birhanu KS, Ketema BH, Zegeye H, Tesfaye G (2013) Case control study on measles outbreak investigation in Abaya District, Southern Ethiopia (Unpublished)

[13] Central Statistical Agency of Ethiopia (CSA) (2005) Ethiopian demographic and health survey

[14] Federal Ministry of Health (MOF). Health sector development program IV 2010/11–2014/15

[15] Lim SS, Stein DB, Charrow A, Murray CJL (2008). Tracking progress towards universal childhood immunization and the impact of global initiatives: a systematic analysis of three-dose diphtheria, tetanus, and pertussis immunization coverage. Lancet.

[16] Central Statistical Agency of Ethiopia (CSA) (2007) National census of Ethiopia. In: Ministry of Finance and Economic Development (ed). Addis Ababa

[17] Lay Armachiho District Health Office (2013) Annual health report of the District Gondar, Ethiopia

[18] Getu D, Fasil T (2005) Biostatistics lecture note for health sciences students. The Carter Center, Addis Ababa, Ethiopia, p 181

[19] Wenjing T, Max P, Forsberg BC (2013). Routine vaccination coverage in low- and middle-income countries: further arguments for accelerating support to child vaccination services. Global Health Action.

